# Fetal Left Ventricle Function Evaluated by Two-Dimensional Speckle-Tracking Echocardiography across Clinical Stages of Severity in Growth-Restricted Fetuses

**DOI:** 10.3390/diagnostics14050548

**Published:** 2024-03-05

**Authors:** Carla Domínguez-Gallardo, Nuria Ginjaume-García, Johana Ullmo, Juan Parra, Ana Vázquez, Mónica Cruz-Lemini, Elisa Llurba

**Affiliations:** 1 Department of Obstetrics and Gynaecology, Institut d’Investigació Biomèdica Sant Pau-IIB Sant Pau, Hospital de la Santa Creu i Sant Pau, Universitat Autònoma de Barcelona, 08025 Barcelona, Spain; cdominguezg@santpau.cat (C.D.-G.); nuriag544@gmail.com (N.G.-G.); jullmo@santpau.cat (J.U.); jparra@santpau.cat (J.P.); ellurba@santpau.cat (E.L.); 2 Women and Perinatal Health Research Group, Sant Pau Biomedical Research Institute (IIB-Sant Pau), 08025 Barcelona, Spain; 3 Maternal and Child Health and Development Network (SAMID), RD16/0022, Instituto de Salud Carlos III, 28029 Madrid, Spain; 4 Applied Statistics Department, Universitat Autònoma de Barcelona, 08025 Barcelona, Spain; ana.vazquez@uab.cat; 5 Primary Care Interventions to Prevent Maternal and Child Chronic Diseases of Perinatal and Developmental Origin Network (RICORS, RD21/0012/0001), Instituto de Salud Carlos III, 28029 Madrid, Spain

**Keywords:** fetal echocardiography, prenatal diagnosis, 2D speckle tracking, strain, fetal growth restriction, fetal cardiac function

## Abstract

Fetal growth restriction (FGR) can result in adverse perinatal outcomes due to cardiac dysfunction. This study used 2D speckle-tracking echocardiography to assess left ventricle (LV) longitudinal strain across FGR severity stages. A prospective longitudinal cohort study measured global (GLS) and segmental LV longitudinal strain in FGR fetuses, with evaluations conducted at various time points. FGR was classified into subtypes based on published criteria using fetal weight centile and Doppler parameters. A linear mixed model was employed to analyze repeated measures and compare Z-score measurements between groups throughout gestational age. The study included 40 FGR fetuses and a total of 107 evaluations were performed: 21 from small for gestational age (SGA), 74 from the FGR stage I, and 12 from the FGR stage ≥ II. The results indicate that SGA and stage I FGR fetuses exhibit higher LV GLS than stages ≥ II. Throughout gestation, SGA and FGR stage I fetuses showed similar behavior with consistently better LV GLS values when compared to FGR stages ≥ II. No significant differences were observed in LV GLS strain behavior between SGA and FGR stage I. In conclusion, all FGRs show signs of early cardiac dysfunction, with severe cases demonstrating significantly a lower LV GLS when compared to mild cases, suggesting deterioration of cardiac dysfunction with progression of fetal compromise.

## 1. Introduction

Fetal growth restriction (FGR) is a condition characterized by impaired fetal growth and development, often resulting from placental insufficiency. FGR poses significant risks to both the fetus and the neonate due to fetal programming, with adverse perinatal outcomes ranging from increased morbidity to long-term health consequences [1,2]. 

In clinical practice, FGR fetuses are classified into different stages of severity based on the sequential decline of fetoplacental Doppler patterns [3]. However, the precise onset and progression of fetal cardiac dysfunction across these stages remain unclear. Understanding the impact of FGR on cardiac function is crucial for optimizing fetal and neonatal management.

The fetal heart of FGR fetuses adapts to placental insufficiency by changing its shape and structure, in order to ensure optimal blood supply to organs. This is called fetal cardiac remodeling, which has been previously reported [4] as changes in cardiac shape towards a more globular heart, characterized by hypertrophy and ventricle dilatation. This cardiac remodeling is usually accompanied by changes in fetal heart function. The assessment of cardiac function in FGR has traditionally relied on conventional echocardiography measures and Doppler evaluation [5,6,7]. However, these parameters have demonstrated to have some limitations and may not be sensitive enough to detect subtle changes in cardiac function during fetal development [7]. Recent advances in echocardiographic techniques, specifically 2D speckle-tracking echocardiography (2D-STE), have provided a novel approach for evaluating myocardial mechanics and strain [8,9,10,11,12,13].

Cardiac strain, measured through 2D-STE, represents deformation of myocardial tissue during the cardiac cycle and provides valuable insights into myocardial performance, enabling a comprehensive assessment of regional and global myocardial function. In the context of FGR, the evaluation of longitudinal endocardial deformation has the potential to detect early signs of cardiac dysfunction [14], due to longitudinal endocardial myofibers being more vulnerable to ischemia [15]. While several studies have investigated cardiac function and strain in FGR fetuses, the existing literature remains limited and fragmented [8,16]. More studies about cardiac function and strain evaluation in FGR are needed to consolidate available evidence, enhance understanding of this subject, and outline future directions for investigation.

In this article, we aim to assess fetal left ventricle (LV) longitudinal strain across clinical stages of severity in FGR, and to evaluate LV strain behavior across gestation, using automated 2D speckle-tracking software. 

## 2. Materials and Methods

### 2.1. Study Population

Our study focused on pregnant women with singleton pregnancies who received care at the Maternal-Fetal Medicine Department of Hospital de la Santa Creu i Sant Pau in Barcelona, Spain, between June 2018 and December 2021. We conducted a prospective enrollment of fetuses with estimated fetal weight (EFW) below the 10th percentile [3]. Gestational age (GA) of all pregnancies was determined based on crown–rump length measured during first trimester ultrasound [17]. Local reference curves were used to calculate the estimated fetal weight and birth weight centiles [18]. 

Distinction between small for gestational age (SGA) and FGR subtypes was made using local reference curves and a previously published classification [3]. For establishing FGR severity stages, Doppler parameters such as pulsatility indices (PI) for umbilical artery (UA), middle cerebral artery (MCA), uterine arteries (UtA), and ductus venosus (DV) were evaluated. Cerebroplacental ratio (CPR) was calculated as the ratio between MCA-PI and UA-PI. The presence of, absence of, or reversed end-diastolic blow flow in UA and DV were also described. SGA was defined as an EFW between 3rd and 10th percentile with normal Doppler evaluations. FGR stage I was defined as an EFW below 3rd percentile or an EFW between 3rd and 10th percentile with CPR and/or MCA-PI below 5th percentile, or UtA-PI over 95th percentile. FGR stage II was classified by an absent diastolic flow in UA and FGR stage III by reversed diastolic flow in UA, absent diastolic flow in DV or DV-PI over 95th percentile. Finally, FGR stage IV was defined when reversed diastolic flow in DV was present. 

Patients with maternal age below 18 years, twin pregnancy and structural or chromosomal anomalies were excluded. Throughout the pregnancy, we collected information on any pregnancy-related conditions that could potentially impact fetal heart remodeling, such as preeclampsia. Data including maternal age at inclusion, body mass index (BMI), race, and parity were recorded. After delivery, we documented perinatal outcomes, including GA at delivery, mode of delivery, birth weight, birth weight percentile and neonatal outcomes. We confirmed that all included patients had a birth weight percentile below the 10th; otherwise, they were excluded from the study. Perinatal mortality was defined as either neonatal death within the first 28 days or intrauterine death. 

Our study protocol (IIBSP-CMQ-2017-99) underwent review and approval by the Ethics Committee of our hospital, and written informed consent was obtained from all participating patients.

### 2.2. Ultrasound Acquisition 

Ultrasound images were obtained using two different systems, Affiniti 70G and EPIQ 7W (Philips Healthcare, Andover, MA, USA). In all cases, fetal routine follow-up was conducted, which included assessments of fetal biometry, fetal echocardiography, and Doppler evaluation. Fetal echocardiography was performed in all examinations conducted from the day of inclusion until delivery. A 9 MHz sector probe (C9-2, Philips Medical Systems, Andover, MA, USA) was utilized to obtain a 4-chamber view of the fetal heart for 2D speckle-tracking evaluation. Image acquisition was adhered to stringent criteria outlined in previously published recommendations [11], and was performed by experienced obstetricians specialized in fetal cardiology imaging and placental dysfunction. Great care was taken to optimize image quality and ensure image acquisition at a frame rate exceeding 80 Hz. 

### 2.3. Analysis Protocol

**Figure 1:**
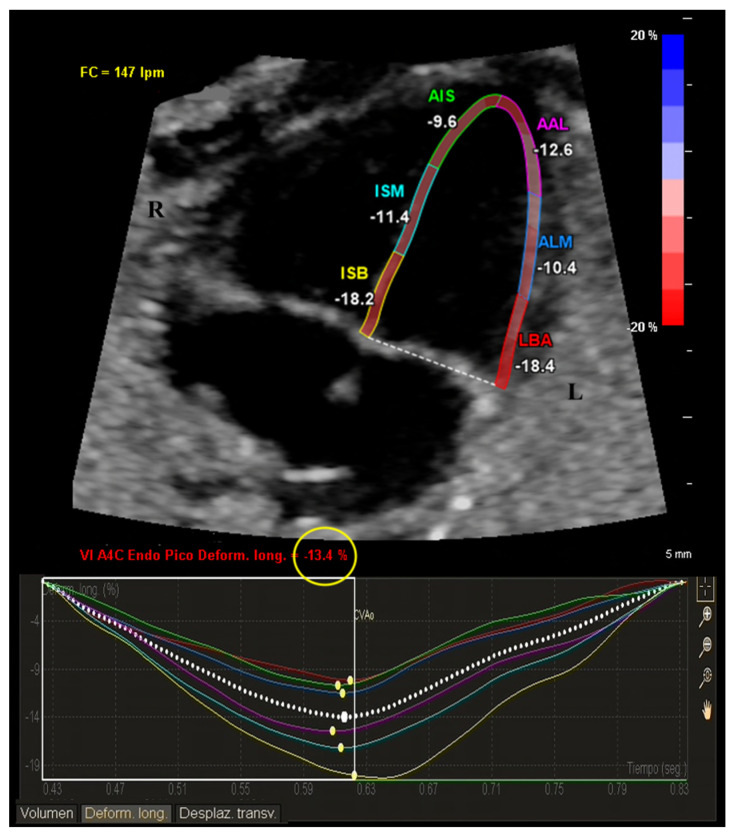
LV longitudinal strain analysis with aCMQ-QLab in FGR stage IV. The software automatically delineated the left-ventricular myocardium, providing LV global longitudinal strain (yellow circle), as well as individual segment measurements; from left to right: basal segment of left-ventricle wall (LBA), middle segment of left-ventricle wall (ALM), apical segment of left-ventricle wall (AAL), basal inter-ventricular septum (ISB), middle interventricular septum (ISM), and apical interventricular septum (AIS). L, left; R, right. Abbreviations differ from the manuscript, due to software language (Spanish).

After confirming an appropriate clip quality, a clip containing 3 or 4 cardiac cycles was selected for offline analysis using the aCMQ-QLab software package (Version 4.7, Philips Medical Systems, Andover, MA, USA). To determine the cardiac cycle manually, a well-established protocol was followed [11]. This involved selecting the endocardial LV border, tracing the LV endocardium, visually inspecting the tracking quality, and making manual corrections if necessary. Subsequently, the software automatically provided global longitudinal strain (GLS) values and segmental strain values for the following six segments (shown in Figure 1): basal interventricular septum (BIS), middle interventricular septum (MIS), apical interventricular septum (AIS), basal segment of the left ventricle wall (BAL), middle segment of the left ventricle wall (MAL), and apical segment of the left ventricle wall (AAL). Additionally, the software provided estimated LV ejection fraction (EF).

### 2.4. Statistical Analysis

To facilitate strain evaluation comparisons between groups, all measurements were normalized into Z-scores (Zs) using previously published reference curves [11]. These Z-score variables were standardized to have a mean of 0 and a standard deviation of 1.

Descriptive analysis was conducted using the IBM SPSS Statistics 26 statistical package. The normal distribution of the variables under study was assessed using the Kolmogorov–Smirnov test. Comparisons between study groups were performed using one-way analysis of variance (ANOVA). Post hoc pairwise comparisons among diagnostic groups were carried out by applying a Bonferroni correction to the *p*-value of each test. Dichotomous variables were analyzed using the χ^2^-test and Fisher’s exact test, with *p* < 0.05 considered statistically significant, and the results are presented as mean ± standard deviation (SD) or percentages (n).

For longitudinal analyses, a linear mixed model was employed to compare the evolution of Z-score measurements between groups throughout gestational age (GA), with subject being treated as the random effect. The explanatory variables in the models included GA, study group, and the interaction between them. The estimated means and standard error (SE) for each model were presented. Post hoc comparisons between groups were obtained. These comparisons were corrected by Tukey method. The estimated means for each group throughout GA were plotted. A significance level of 0.05 was set for all tests conducted. This statistical analysis was performed using SAS software v9.4 (SAS Institute Inc., Cary, NC, USA).

## 3. Results

### 3.1. Basic Characteristics of the Study Population 

Our study initially included 44 women diagnosed with FGR fetuses, with an average gestational age at inclusion of 28.2 weeks, but four cases, in which delivery data were unavailable due to delivery occurring in another hospital, were excluded. Due to the small number of fetuses in severe stages in which we were able to perform follow-up, we grouped these, analyzing them as three groups: 5 SGA, 30 FGR stage I, and 5 FGR stage ≥ II fetuses.

Table 1 presents the characteristics of the study population. There were no significant differences between groups regarding maternal characteristics. As expected, the GA at delivery was earlier in the FGR groups, with vaginal delivery being more frequent in mild cases, and cesarean section in severe FGR stages, due to fetal indication. There was one case of intrauterine death at 27.4 w in FGR stage I group and two neonatal deaths, in a stage II and a stage IV fetus. Only one SGA case required neonatal intensive care unit admission in contrast to 22.2% of FGR stage I and all severe cases, as expected.

### 3.2. Fetal Ultrasound Assessment

**Figure 2:**
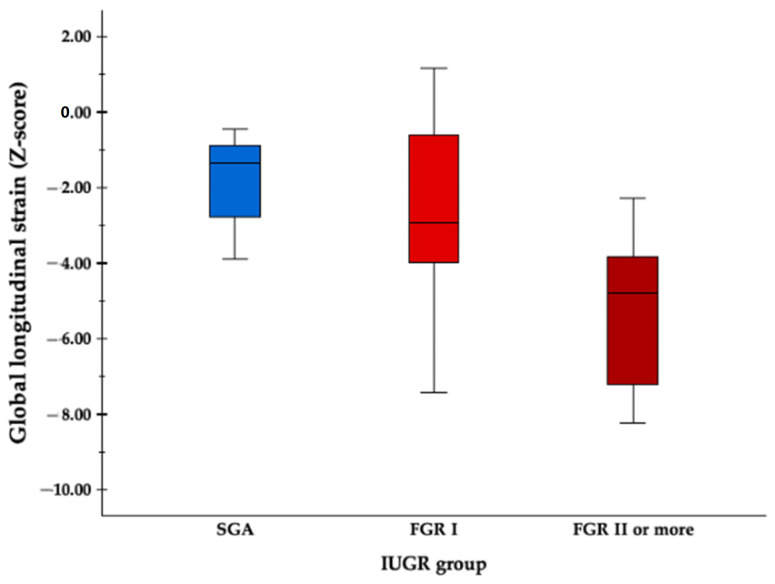
Boxplot for mean LV GLS values in different FGR severity stages. Progressive lower mean strain values are seen as fetal condition deteriorates. SGA, small for gestational age; FGR, fetal growth restriction.

Longitudinal follow-up was performed in all forty cases of FGR fetuses since inclusion, with an average of 2.5 evaluations per fetus, obtaining a total of 107 clips. A total of 21 clips belonged to SGA fetuses, and 86 to FGR fetuses: 74 stage I and 12 stages ≥ II. The frequency of follow-up varied depending on the degree of FGR severity, with some severe cases needing monitoring every 2–3 days, while mild cases were followed up every 2–3 weeks. Table 2 shows LV strain parameters and fetoplacental Doppler at first ultrasound evaluation. The Doppler parameters showed significantly worse values across stages of FGR severity, as expected. The strain measurements were feasible in 100% of acquisitions. The mean frame rate was 104 ± 19 fps for 2D-STE acquisition. A statistically significant lower GLS was observed at diagnosis in severe FGR cases when compared to mild cases (Figure 2). The LV segmental strain showed lower strain values in severe FGR cases, especially in apical segments (AAL and AIS), although no statistically significant differences were observed.

**Figure 3:**
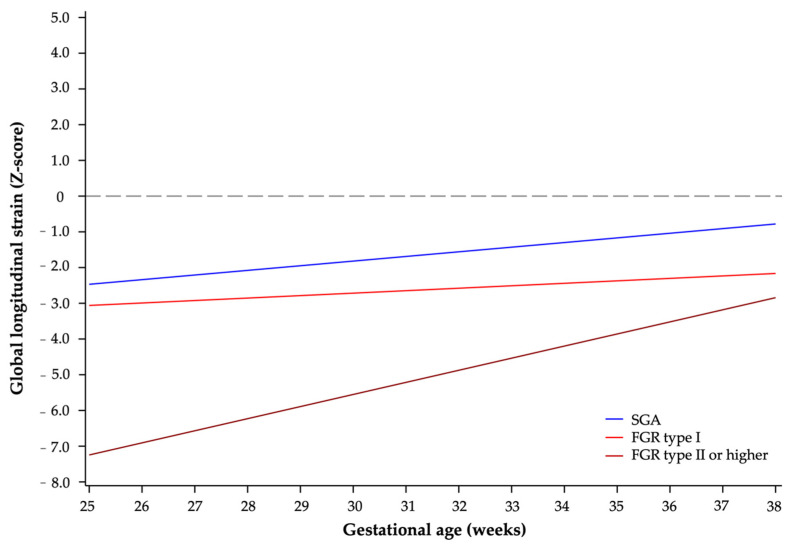
Differences in LV GLS behavior between FGR groups across gestational age. Lower mean strain values are seen with severe FGR, which are maintained throughout gestation. GLS, global longitudinal strain; FGR, fetal growth restriction; SGA, small for gestational age.

Table 3 shows longitudinal analyses for LV strain values across the different FGR groups. For this analysis, FGR groups II, III, and IV were also included as a single entity because of the small number of cases for each. Early cardiac dysfunction was observed even in mild cases since diagnosis, with a trend towards normalization as gestation progressed in the SGA group. A statistically significant lower LV GLS strain was found in severe FGR cases (FGR ≥ II) when compared to SGA and FGR stage I across gestational age (Figure 3). However, there were no significant differences in LV GLS behavior between SGA and FGR stage I.

When evaluating segmental LV strain behavior, we observed that apical segments seemed to be the most affected ones by the hypoxic environment caused by placental insufficiency, showing lower strain values in all FGR cases. A significant correlation in AAL segment was predominantly observed, showing constantly worse strain values across different stages of FGR severity. Septal (BIS, MIS, and AIS) and the other free lateral wall segments (BAL and MAL), although not statistically significant, showed a different trend. Septal segments presented lower strain values in severe FGR cases, whereas free lateral wall segments BAL and MAL, showed higher strain values. Finally, the ejection fraction showed a progressive worsening in severe FGR group as gestational age advanced, whereas SGA and mild FGR cases were born with normal ejection fraction (Supplementary Figure S1). 

## 4. Discussion

The objective of this study was to compare LV strain behavior among different clinical severity stages of FGR, by means of 2D-STE [3,18]. To the best of our knowledge, this is the first study to compare fetal LV longitudinal strain behavior between FGR stages of severity, throughout gestational age. Our research shows the progression of cardiac dysfunction in different FGR groups classified by Doppler stages of severity, used in our clinical setting, suggesting that subclinical cardiac dysfunction is an initial and advancing occurrence in cases of FGR. Our study observed that global LV deformation decreases across FGR severity stages, and we also described different LV longitudinal strain behavior across gestational age in severe FGR cases when compared to mild cases.

Differences in longitudinal strain in FGR have been previously reported [14,16], even in the absence of Doppler abnormalities [8], highlighting poorer ventricle contractility in FGR when compared to adequate for gestational age fetuses (AGA) and demonstrating a subclinical cardiac dysfunction in these fetuses, similarly to that described by other studies [4,19]. Previously published studies have also demonstrated the efficacy of different echocardiographic tools [4,5,8,20,21] in assessing cardiac dysfunction associated with FGR. Due to the technical limitations these tools have, recent studies focused their interest on the evaluation of fetal ventricular strain by 2D-STE [11,13,14,22,23]. In this context, speckle-tracking has emerged as promising solution, offering a semi-automated analysis that reduces intra- and interobserver variability [11,13]. Nonetheless, conflicting results have been reported in some studies, primarily due to the existence of multiple commercialized programs with different acquisition protocols and ultrasound equipment, making results non-comparable [16,24].

Our research group has previously published data on the feasibility, reproducibility, and establishment of normal gestational age-adjusted reference ranges using the aCMQ-Qlab software package [11]. This software is widely utilized postnatally in our clinical setting, enabling longitudinal surveillance of strain without intervendor variability, and facilitating the monitoring of fetal cardiac conditions pre- and post-birth. This standardized approach eliminates discrepancies associated when evaluating the same parameter using different ultrasound equipment or software before and after birth.

Our study demonstrates FGR is associated with abnormal LV strain values, showing poorer LV GLS in more severe FGR stages. This finding is present from FGR diagnosis and remains until delivery. A trend to increase LV GLS values as gestational age advances was observed, but only SGA cases were born with an almost normal LV GLS, whereas FGR stages I and ≥II fetuses were born with marked cardiac dysfunction. Although these differences were not statistically significant, probably due to a small number of SGA cases, it could be hypothesized that the presence of placental insufficiency and chronic hypoxia, absent in SGA cases, plays an important role in fetal cardiac function and remodeling, leading to newborns with subclinical cardiac dysfunction. 

This finding is in line with previously described cardiac remodeling occurring in FGR fetuses [4]. Placental insufficiency leads to fetal hypoxia, causing a decrease in myocardial contractile force (which decreases linearly as local perfusion decreases [15]), and simultaneously decreasing deformation. In cases of chronic hypoperfusion, changes in tissue elasticity may occur, leading to fibrosis and necrosis, causing further decline in deformation, adversely affecting fetal cardiac function [4,15]. 

Differences in segmental deformation are also evident in our study. In the SGA group all LV segments showed a trend towards better and normal values as GA advanced. FGR stage I showed similar behavior, whereas most notable differences were observed in FGR stages II and above. When evaluating segmental LV strain behavior, we observed that apical segments seem to be the most affected by the hypoxic environment caused by placental insufficiency, showing lower strain values in all FGR cases. We could only demonstrate a statistically significant lower AAL strain in severe FGR cases across gestation, probably due to the small number of cases with severe conditions, with a similar trend observed in septal segments (BIS, MIS, and AIS). On the other hand, free wall segments BAL and MAL behaved differently, slightly increasing their deformation. We hypothesize this could be explained by an attempt by these segments to compensate lower deformation of neighboring ones. Segmental analysis is challenging, especially in pathological conditions [15], due to integration of the segment into the whole muscle and interaction with other segments. Despite this limitation, our findings lead us to the conclusion that apical segments may exhibit heightened sensitivity to placental ischemia and wall stress, ultimately reducing their deformation and contributing to cardiac remodeling in affected fetuses [15]. 

Our study aligns with prior research, which has demonstrated fetal cardiac remodeling in cases of FGR, resulting in reduced longitudinal deformation [2] and alterations in cardiac shape, such as a more globular and hypertrophic phenotype [25,26]. Our study provides strong evidence to support the idea that subclinical cardiac dysfunction is an early and progressive occurrence in severe cases of FGR, contributing to current understanding of cardiac function and strain evaluation in this condition. Previously published studies have reported on the presence of blood markers of cardiac dysfunction [27,28] in FGR cases and also correlation between abnormal echocardiographic parameters and risk of adverse outcomes or perinatal death [19,29]. Importantly, these changes tend to persist into postnatal life [2,25].

The results of our study present compelling evidence indicating that cardiac dysfunction is an early occurrence in fetal growth restriction, and may be evaluated by 2D STE. This implies that subclinical cardiac dysfunction can be observed in fetuses experiencing FGR, even in cases with mild degrees of Doppler deterioration, and followed up during pregnancy. Severity of the fetal condition appears to be proportional to the magnitude of cardiac dysfunction, showing a good correlation with Doppler evaluation, widely used in clinical practice. Integrating ultrasound-based evaluation of cardiac function into clinical practice may have the potential to enhance accuracy of short-term fetal compromise prediction. Including 2D-STE in this assessment could improve fetal follow-up and help in decision making in cases with risk of cardiac dysfunction. 

Our study has various strengths. It is a prospective study, performed in a very well selected population and very well classified. The data were rigorously collected regarding all evaluations, and regarding delivery outcomes, verifying that fetuses were correctly classified for analysis. It is essential to also acknowledge several limitations within our study. First, the use of commercial software may yield different results owing to variations in algorithms [24]. Therefore, the generalizability of our findings to other software vendors is uncertain. Furthermore, the novel software used in our study only accommodates LV analysis in a four-chamber view, limiting its clinical applicability for certain fetal conditions, even though efforts are underway to validate RV evaluation. Regarding deformation analysis, in our study, we only analyzed longitudinal ventricle deformation. Although it is crucial to recognize that assessment of circumferential and axial strain is ideal, longitudinal myocardial fibers are typically the initial site of involvement in most cardiac conditions [15]. Also, the fetal heart is quite small, and at this moment, the acquisition of short-axis views required for other strain evaluations is limited. Lastly, despite a substantial number of examinations performed in our study, the sample size within the most severe subgroups may limit the drawing of robust conclusions concerning our outcomes. Consequently, further studies are warranted to elucidate strain behavior in cases of severe hypoxia.

## 5. Conclusions

Early on, upon diagnosis, FGR fetuses show signs of subclinical cardiac dysfunction. In SGA cases, cardiac function appears to remain stable with a trend towards normalization, while FGR severe cases (FGR ≥ II stage) maintain cardiac dysfunction. Therefore, 2D-STE could help in the monitoring and follow-up of these fetuses, improve risk stratification, and aid in management of long-term outcomes of affected infants.

## Figures and Tables

**Table 1:** Basic characteristics of the study population.

Variable	SGA (n = 5)	FGR I (n = 30)	FGR ≥ II (n = 5)	*p*
Clinical characteristics				
Maternal age, years	35 ± 6	34 ± 6	31 ± 2	0.473
Caucasian	100 (5)	80 (24)	40 (2)	0.195
Latin-American	0 (0)	10 (3)	40 (2)	0.195
Body mass index at inclusion, kg/m^2^	25.8 ± 5.8	23.6 ± 4.8	25.2 ± 3.6	0.564
Nulliparity	100 (5)	53.3 (16)	40 (2)	0.104
Pregnancy outcome				
Neonatal male sex	20 (1)	33.3 (10)	100 (5)	0.012
Preeclampsia	20 (1)	10 (3)	20 (1)	0.710
GA at delivery	39.2 ± 1.4	37.1 ± 2.8	29.7 ± 3.1 *^†^	<0.001
Vaginal delivery	80 (4)	66.6 (20)	0 (0)	0.032
Caesarean delivery	20 (1)	33.3 (10)	100 (5)	0.032
Birth weight, g	2577 ± 210	2215 ± 488	1233 ± 464 *^†^	<0.001
Birth weight centile APGAR 1′ APGAR 5′ Neonatal intensive care unit admission	3 ± 1 8 ± 2 9 ± 2 25 (1)	2 ± 3 8 ± 1 9 ± 2 22 (6)	1 ± 2 6 ± 2 8 ± 1 100 (5)	0.463 0.071 0.473 0.003
Perinatal mortality	0 (0)	3.3 (1)	40 (2)	0.012

Data are shown as mean ± standard deviation or percentage (n). * *p* value < 0.05 compared to SGA group; ^†^ *p* value < 0.05 compared to FGR I group.

**Table 2:** Doppler and LV strain Z-scores in the different FGR groups at first evaluation.

Variable	SGA (n = 5)	FGR I (n = 30)	FGR ≥ II (n = 5)	*p*
Fetoplacental US				
GA at US (weeks)	31.2 ± 0.6	30.9 ± 3.5	28.7 ± 2.6	0.317
EFW (g)	1433 ± 75	1353 ± 531	943 ± 365	0.171
EFW (centile)	5 ± 2	2 ± 2	1 ± 2 *	0.027
UA-PI (Zs)	0.09 ± 0.67	0.62 ± 1.20	3.93 ± 1.90 *^†^	<0.001
MCA-PI (Zs)	−0.17 ± 0.91	−0.20 ± 1.13	−1.56 ± 0.59 *^†^	0.035
CPR (Zs)	−0.64 ± 1.04	−1.02 ± 1.15	−3.18 ± 0.50 *^†^	0.002
UtA-PI (Zs)	0.75 ± 0.67 ^†^	1.93 ± 1.60	3.53 ± 0.78 *	0.008
LV strain values (Zs)				
GLS	−1.85 ± 1.44	−2.54 ± 2.06	−5.27± 2.43 *^†^	<0.05
BAL	−0.49 ± 1.42	−0.08 ± 1.23	−1.98 ± 3.05 ^†^	<0.05
MAL	0.20 ± 0.78	0.34 ± 0.90	1.37 ± 0.94	0.053
AAL	−1.04 ± 1.36	−1.90 ± 2.10	−2.74 ± 2.45	0.369
BIS	0.31 ± 0.85	1.15 ± 1.17	−0.34 ± 0.68	0.093
MIS	0.62 ± 1.97	−0.29 ± 1.40	−1.20 ± 0.89	0.324
AIS	−2.29 ± 2.33	−1.92 ± 2.75	−5.04 ± 2.64	0.065
EF	−0.43 ± 1.39	−0.52 ± 2.32	−1.90 ± 1.99	0.410

Data are shown as mean ± standard deviation. Doppler and strain parameters are expressed in Z-scores (Zs). * *p* value < 0.05 compared to SGA group; ^†^ *p* value < 0.05 compared to FGR I group; AAL, apical segment of left-ventricle wall; AIS, apical interventricular septum; BAL, basal segment of left-ventricle wall; BIS, basal inter-ventricular septum; CPR, cerebroplacental ratio; EF, ejection fraction; EFW, estimated fetal weight; FGR, fetal growth restriction; GA, gestational age; GLS, global longitudinal strain; LV, left ventricle; MAL, middle segment of left-ventricle wall; MCA, middle cerebral artery; MIS, middle interventricular septum; PI, pulsatility index; SGA, small for gestational age; UA, umbilical artery; US, ultrasound; UtA, uterine artery.

**Table 3:** Global differences in least squares mean between groups.

Differences in Least Squares Means			
LV Strain Evaluation (Zs)	Group	Estimate (SE)	Adj *p*
GLS Zs			
	SGA vs. FGR I	1.03 (0.56)	0.167
	SGA vs. FGR ≥ II	3.27 (0.96)	0.003
	FGR I vs. FGR ≥ II	2.24 (0.85)	0.029
BIS Zs			
	SGA vs. FGR I	0.13 (0.33)	0.913
	SGA vs. FGR ≥ II	0.17 (0.54)	0.944
	FGR I vs. FGR ≥ II	0.04 (0.48)	0.996
MIS Zs			
	SGA vs. FGR I	0.64 (0.37)	0.209
	SGA vs. FGR ≥ II	1.57 (0.64)	0.046
	FGR I vs. FGR ≥ II	0.93 (0.57)	0.247
AIS Zs			
	SGA vs. FGR I	0.95 (0.74)	0.409
	SGA vs. FGR ≥ II	2.77 (1.24)	0.075
	FGR I vs. FGR ≥ II	1.82 (1.11)	0.238
BAL Zs			
	SGA vs. FGR I	−1.04 (0.41)	0.035
	SGA vs. FGR ≥ II	−0.29 (0.69)	0.909
	FGR I vs. FGR ≥ II	0.76 (0.62)	0.442
MAL Zs			
	SGA vs. FGR I	−0.03 (0.22)	0.992
	SGA vs. FGR ≥ II	−0.66 (0.38)	0.203
	FGR I vs. FGR ≥ II	−0.64 (0.34)	0.159
AAL Zs			
	SGA vs. FGR I	1.46 (0.49)	0.012
	SGA vs. FGR ≥ II	3.15 (0.85)	0.001
	FGR I vs. FGR ≥ II	1.68 (0.76)	0.078
EF Zs			
	SGA vs. FGR I	0.46 (0.49)	0.623
	SGA vs. FGR ≥ II	3.12 (0.90)	0.003
	FGR I vs. FGR ≥ II	3.59 (0.80)	0.000

Data are shown as mean estimated difference ± standard error (SE). Strain parameters are expressed in Z-scores (Zs). *p* value < 0.05. AAL, apical segment of left-ventricle wall; AIS, apical interventricular septum; BAL, basal segment of left-ventricle wall; BIS, basal interventricular septum; EF, ejection fraction; FGR, fetal growth restriction; GLS, global longitudinal strain; LV, left ventricle; MAL, middle segment of left-ventricle wall; MIS, middle interventricular septum; SGA, small for gestational age.

## Data Availability

The data that supports findings of this study are not publicly available, due to their containing information that could compromise the privacy of research participants, but may be available upon request.

## Supplementary Materials

The following supporting information can be downloaded at: https://www.mdpi.com/article/10.3390/diagnostics14050548/s1, Figure Supplementary S1: Differences in LV strain Z-scores behavior between FGR groups across gestational age.
