# Supplementary material for: Fetal Left Ventricle Function Evaluated by Two-Dimensional Speckle-Tracking Echocardiography across Clinical Stages of Severity in Growth-Restricted Fetuses
Source: Diagnostics (Basel). 2024 Mar 5;14(5):548. doi: 10.3390/diagnostics14050548 (PMC10931078; doi:10.3390/diagnostics14050548)

Supplementary Figure S1. Differences in LV segmental strain behavior between FGR groups across gestational age. FGR, fetal growth restriction; SGA, small for gestational age.

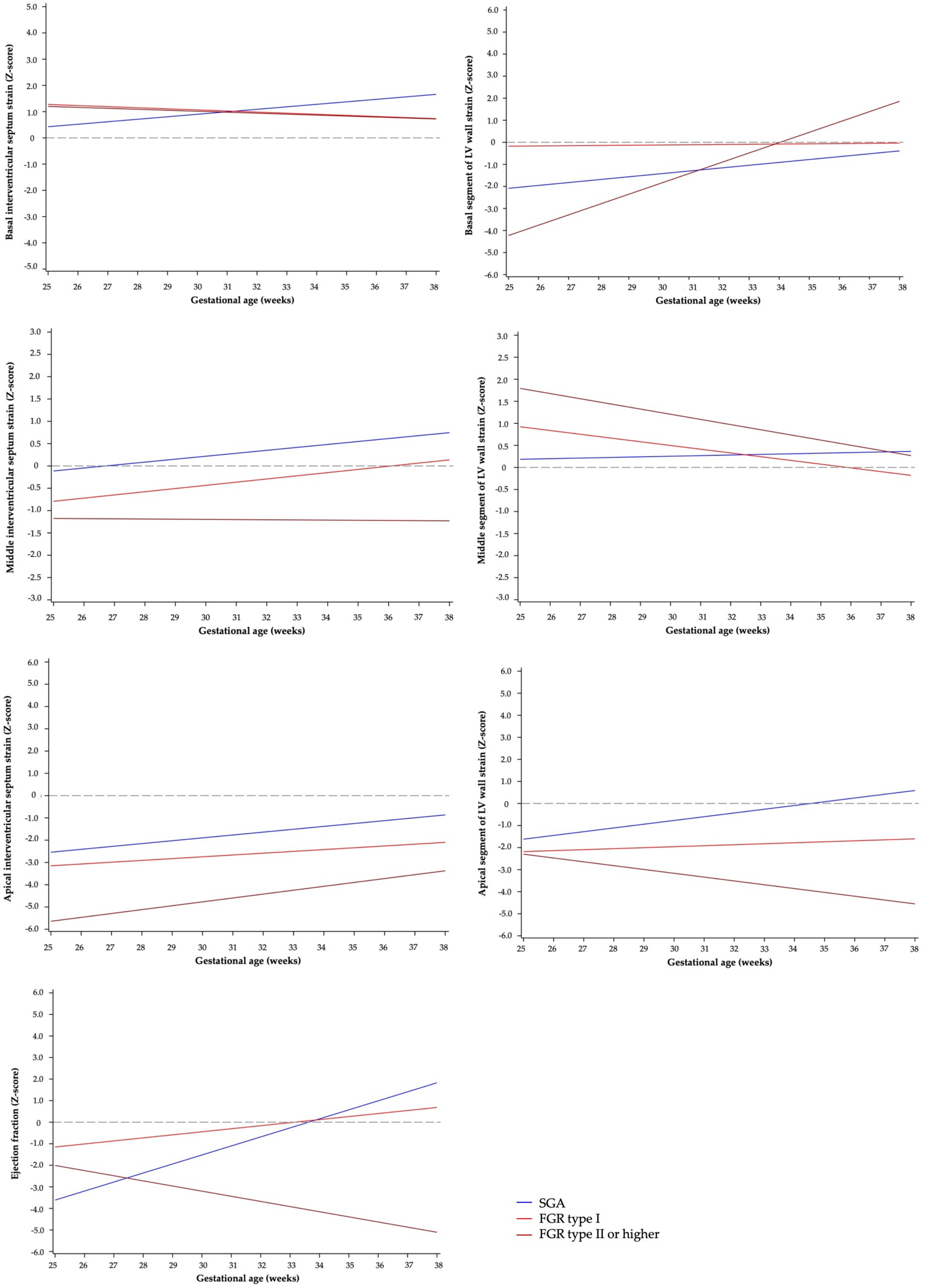

Supplement: Supplementary file 1 [file diagnostics-14-00548-s001.zip › diagnostics-2874813-supplementary.pdf]
